# Supplementary material for: Pseudomonas PS01 Isolated from Maize Rhizosphere Alters Root System Architecture and Promotes Plant Growth
Source: Microorganisms. 2020 Mar 26;8(4):471. doi: 10.3390/microorganisms8040471 (PMC7232511; doi:10.3390/microorganisms8040471)
Supplement: Supplementary file 1 [file microorganisms-08-00471-s001.pdf]

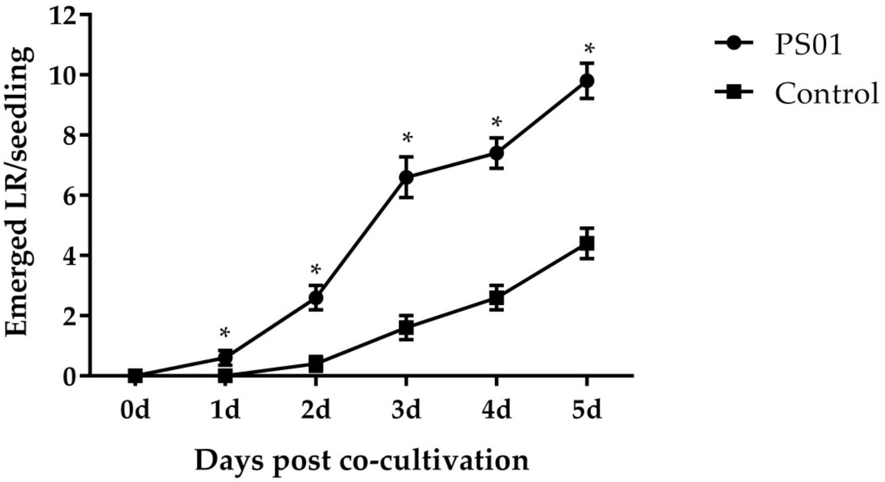

Figure S1 a. Time course of LR initiation in response to PS01. At time point 0 d, bacteria were inoculated on the plates. The values represent means ± SD (n = 15). Asterisks indicate statistically significant differences compared with untreated roots (Student's t test; $p < 0.05$).

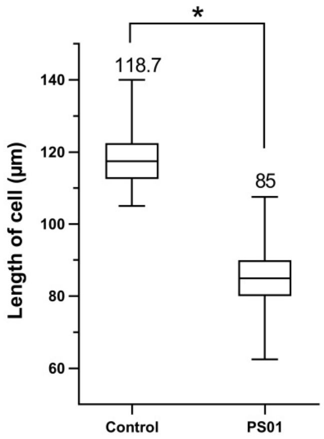

Figure S1 b. Inhibition of root cell elongation in differentiation zone by PS01. Length of cells were observed and measured under a 40 X microscope. Bars represent average lengths ± SD of 75 cells in each developmental zone (n = 15). Asterisks indicate statistically significant differences compared with non-inoculated roots (Student's t test; $p < 0.05$).

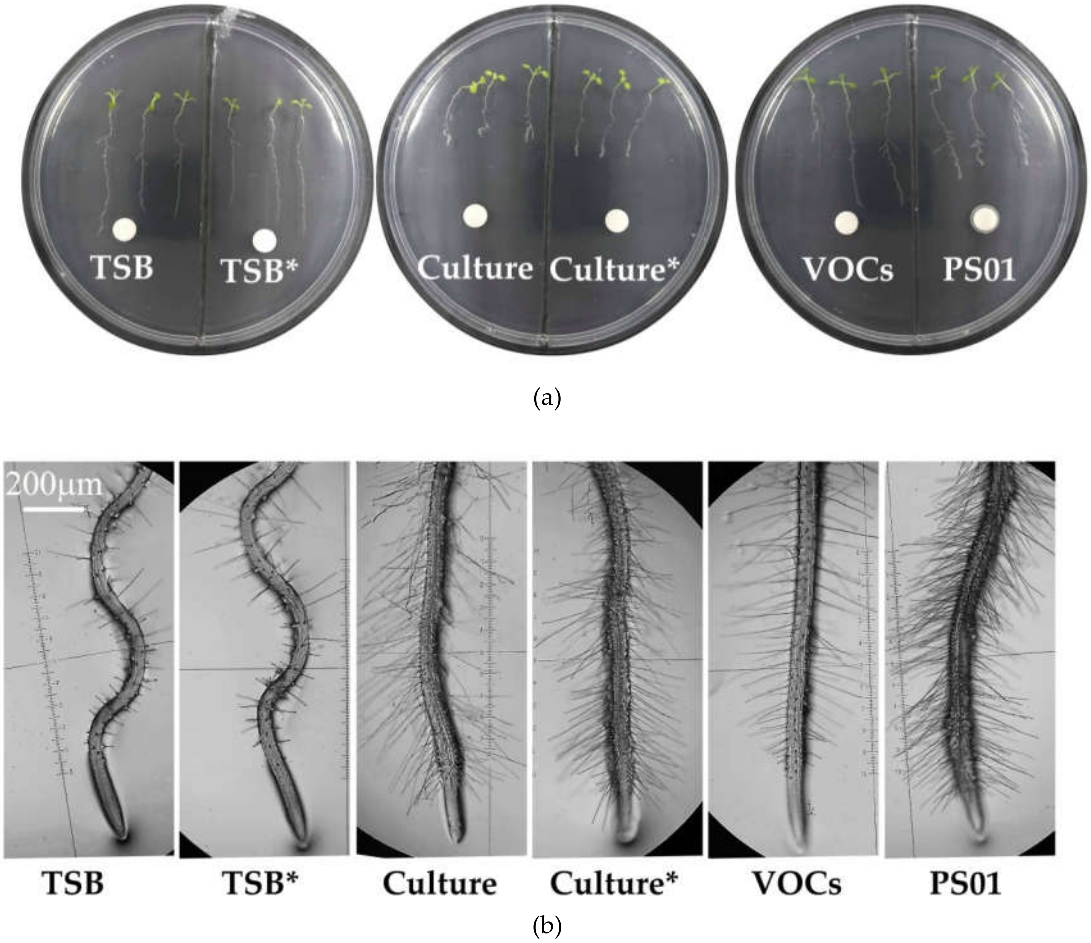

Figure S2. Different secreted metabolites produced by PS01 implicated in RSA of *Arabidopsis*. (a) Split plate assay; (b) Effect of VOCs blend and diffusible compounds on RH development. Photos were taken after 7 days of inoculation.

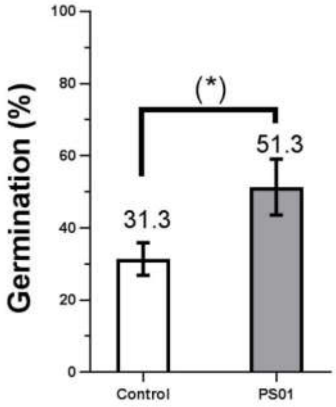

Figure S3. *Pseudomonas* PS01 improves germination rate in *Zea mays*. The germination rate was evaluated after 2 days incubation (100 seeds). Bars represent average values ($n = 3$). Asterisks indicate statistically significant differences compared with non-inoculated roots (Student's t test; $p < 0.05$)

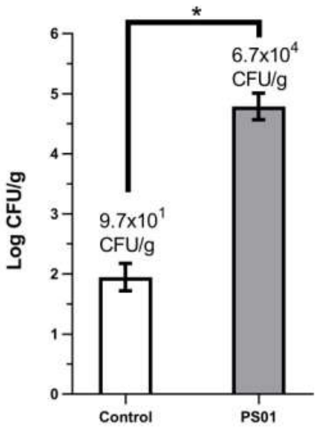

Figure S4. Root colonization of maize plants by PS01. The number of *Pseudomonas* sp. per gram of root was determined at 30 th day. Bars represent average of log CFU g^{-1} ± SD (n = 15). Asterisks indicate statistically significant differences compared with non-inoculated roots (Student's t test; $p < 0.05$)

Table S1. Accession number, name, description, and primer sequences used for quantitative RT-PCR [43]

AGI Code	Name	Description	Fw/Rv primers
At3 g07390	AIR12	Auxin-responsive protein	5′-TGAATCAGGTATGGCAGATCGG-3′(F)<br>5′-TGAAACTCAACACACGGTGGG-3′(R)
At5 g54510	DFL1	IAA-amido synthase that conjugates Ala, Asp, Phe, and Trp to auxin	5′-CACCAAAGATCGCAGCTTTG-3′(F)<br>5′-ATCATCTGCGTTCGTGGTCAC-3′(R)
At1 g56010	NAC1	Transcription factor involved in shoot apical meristem formation and auxin mediated Lateral root formation	5′-GCCTCCGGGATTCAGATTTCA-3′(F)<br>5′-TCAGGACAAGAGGTGGTCGATG-3′(R)
At2 g38120	AUX1	Auxin permease	5′-AAGGGCTTTGGCTAGATTGCC-3′(F)<br>5′-CAAGAAGAGCACCGACAGCG-3′(R)
At1 g04240	IAA3/SHY2	Regulates multiple auxin responses in roots, induced by IAA	5′-GCCAAAGGTTTAGGCTGTGGTG-3′(F)<br>5′-TAGATCTTGCCCGAGAAACCCG-3′(R)
At1 g73590	PIN1	Auxin efflux carrier involved in shoot and root development	5′-CGCTTCAGAGTTCAAGAAACCC-3′(F)<br>5′-CACAGCTTCTCCAGGACCAAAG-3′(R)
At5 g57090	PIN2/EIR1	Auxin efflux carrier	5′-CCAATGTTCACGGGGTCAACG-3′(F)<br>5′-GAAGCACTCGAACTCCACACG-3′(R)
At5 g20960	AAO1	Arabidopsis aldehyde oxidase 1 with the specificity for indole-3-acetaldehyde	5′-TGTGGCGAAGGTGGTTGTGG-3′(F)<br>5′-GCTGCGAGGAGGTGGATGAG-3′(R)
At5 g05730	ASA1	Anthranilate synthase alpha subunit 1	5′-GCGTTGGTCGTTATAGCGTTG-3′(F)<br>5′-TGGGATCTCCATTGGATCTTCG-3′(R)
At3 g54640	TSA1	Tryptophan synthase alpha chain	5′-CGTTGGAGAGGGGAACAAACC-3′(F)<br>5′-TTGGTGTGGTTGGTGTAGTGAG-3′(R)
At5 g43890	YUCCA5	Flavin mono-oxygenase	5′-TGTGTCCAGTCTGCTCGATACG-3′(F)<br>5′-CCCGTCGCCACCACTAACC-3′(R)
At4 g39960	CYP79 B2	Cytochrome P450 that is involved in tryptophan metabolism	5′-CCATGCAGAGACAACAGAAACC-3′(F)<br>5′-AGGTGCTAAAGGACGATGTTTC-3′(R)
At4 g31500	CYP83 B1	Cytochrome P450 mono-oxygenase 83 B1	5′-ACGAGACGCAAGCACTTTTGG-3′(F)<br>5′-GGGCGGTTAGGGTCAAGAGTC-3′(R)
At2 g04400	IGPS	Indole-3-glycerol-phosphate synthase	5′-GGACCATTTGAGTTGAGATTGC-3′(F)<br>5′-CCTAAGAGCCCCAACAAAATCC-3′(R)

Table S2. Gene annotations of PS01 genome related to its IAA- and VOCs-producing ability.

Trait	Contig ID	Peptide ID	Gene annotation	KO/gene ID	KEGG gene name	EC number
IAA biosynthesis TAM pathway	NODE_382	fig|286.823.peg.2319	Tryptophan decarboxylase	K01593	DDC, TDC	4.1.1.28
	NODE_30	fig|286.823.peg.1939	monoamine oxidase	k00274	MAO, aofH	1.4.3.4
	NODE_446	fig|286.823.peg.2840	aldehyde dehydrogenase (NAD+)	K00128	ALDH	1.2.1.3
IAA biosynthesis IAM pathway	NODE_157	fig|286.823.peg.429	Tryptophan 2-monooxygenase	K00466	iaaM	1.13.12.3
	NODE_16	fig|286.823.peg.693	amidase	K01426	amiE	3.5.1.4
	NODE_382	fig|286.823.peg.2179	amidase	K01426	amiE	3.5.1.4

(R)-2-Acetoin	NODE_101	fig|286.823.peg.62	acetolactate synthase I/II/III large subunit	K01652	ilvB, ilvG, ilvI	2.2.1.6
	NODE_152	fig|286.823.peg.367	acetolactate synthase I/II/III large subunit	K01652	ilvB, ilvG, ilvI	2.2.1.6
	NODE_293	fig|286.823.peg.1591	acetolactate synthase I/II/III large subunit	K01652	ilvB, ilvG, ilvI	2.2.1.6
	NODE_76	fig|286.823.peg.3854	acetolactate synthase I/II/III large subunit	K01652	ilvB, ilvG, ilvI	2.2.1.6
	NODE_76	fig|286.823.peg.3855	acetolactate synthase I/III small subunit	K01653	ilvH, ilvN	2.2.1.6
	NODE_209	fig|286.823.peg.1000	(R,R)-butanediol dehydrogenase / meso-butanediol dehydrogenase / diacetyl reductase	K00004	BDH, butB	1.1.1.-
2,3-butanediol	NODE_209	fig|286.823.peg.1000	(R,R)-butanediol dehydrogenase / meso-butanediol dehydrogenase / diacetyl reductase	K00004	BDH, butB	1.1.1.-
						1.1.1.4 1.1.1.303
4-hydroxybenzoate	NODE_446	fig|286.823.peg.2897	chorismate-pyruvate lyase	K03181	ubiC	4.1.3.40
